# A survey of transcriptome complexity using PacBio single-molecule real-time analysis combined with Illumina RNA sequencing for a better understanding of ricinoleic acid biosynthesis in *Ricinus communis*

**DOI:** 10.1186/s12864-019-5832-9

**Published:** 2019-06-06

**Authors:** Lijun Wang, Xiaoling Jiang, Lei Wang, Wei Wang, Chunling Fu, Xingchu Yan, Xinxin Geng

**Affiliations:** 1 Oil Crops Research Institute of the Chinese Academy of Agricultural Sciences/Key Laboratory of Biology and Genetic, Improvement of Oil Crops, Ministry of Agriculture, Wuhan, China; 2 Applied Biotechnology Center, Wuhan University of Bioengineering, Wuhan, China; 3 College of Life Science and Technology, Henan Institute of Science and Technology/Collaborative Innovation Center of Modern Biological Breeding, Xinxiang, China

**Keywords:** *Ricinus communis*, Full-length transcriptome, Illumina RNA sequencing, Ricinoleic acid biosynthesis, Key enzymes

## Abstract

**Background:**

*Ricinus communis* is a highly economically valuable oil crop plant from the spurge family, Euphorbiaceae. However, the available reference genomes are incomplete and to date studies on ricinoleic acid biosynthesis at the transcriptional level are limited.

**Results:**

In this study, we combined PacBio single-molecule long read isoform and Illumina RNA sequencing to identify the alternative splicing (AS) events, novel isoforms, fusion genes, long non-coding RNAs (lncRNAs) and alternative polyadenylation (APA) sites to unveil the transcriptomic complexity of castor beans and identify critical genes related to ricinoleic acid biosynthesis. Here, we identified 11,285 AS-variants distributed in 21,448 novel genes and detected 520 fusion genes, 320 lncRNAs and 9511 (APA-sites). Furthermore, a total of 6067, 5983 and 4058 differentially expressed genes between developing beans of the *R. communis* lines 349 and 1115 with extremely different oil content were identified at 7, 14 and 21 days after flowering, respectively. Specifically, 14, 18 and 11 DEGs were annotated encoding key enzymes related to ricinoleic acid biosynthesis reflecting the higher castor oil content of 1115 compared than 349. Quantitative real-time RT-PCR further validated fifteen of these DEGs at three-time points.

**Conclusion:**

Our results significantly improved the existed gene models of *R. communis*, and a putative model of key genes was built to show the differences between strains 349 and 1115, illustrating the molecular mechanism of castor oil biosynthesis. A multi-transcriptome database and candidate genes were provided to further improve the level of ricinoleic acid in transgenic crops.

**Electronic supplementary material:**

The online version of this article (10.1186/s12864-019-5832-9) contains supplementary material, which is available to authorized users.

## Background

*Ricinus communis* (*R. communis*) L., is a highly economically valuable oil plant from the spurge family, Euphorbiaceae, and arguably one of the non-edible oil crops with the highest application value. Recently, with the looming shortage of non-renewable resources such as oil and natural gas, castor oil has attracted worldwide attention as an alternative renewable resource [1]. However, the previously reported reference genomes are incomplete and had limited annotation and structural information. Although the sequence information of several species obtained through short-read sequencing has been published in recent years [2–4], the knowledge on full-length (FL) sequences of mRNAs remains limited, especially in *R. communis*. FL transcripts can significantly improve the accuracy of genome annotation/ assembly and transcriptome information, which helps to identify full length isoforms of a gene, alternative splicing (AS) events, long non-coding RNAs (lncRNAs) and alternative polyadenylation (APA) sites [5].

Next-generation sequencing (NGS; i.e., Illumina RNA sequencing) can generate digital data on gene expression without the limits of predesigned probes [6], and has been conducted not only to create the reference genomes such as in *Arabidopsis* [7], rice [8], maize [9], but also provides the *de-novo* assembly for many organisms in the past including animals and plants [10, 11]. The results of incomplete (ranging between 1 and 2 kb, including a methylated cap at the 5′ end and poly-A tails at the 3′ end) and low-quality transcripts obtained through Illumina RNA sequencing limit the scope of analysis of alternative splicing variants and corrected annotation [12]. However, the advent of Pacific Biosciences (PacBio) single-molecule long read isoform sequencing (SMRT-seq) technology has enabled us to obtain long-read or full-length transcriptomes, which allows the collection of large-scale long-read transcripts with complete coding sequences and characterization of gene families (the average read length of PacBio SMRT-seq is > 10 kb and the real length can be up to 60 kb) [13–16]. However, it was reported that SMRT-seq provided inaccurate information on genes, less coverage of genes led to the high error rate, which could be corrected using Illumina RNA sequencing reads and circular-consensus (CCS) reads [17]. Recently, the strategy of combining SMRT-seq and Illumina RNA-seq has been applying to generate comprehensive information, detect more gene isoforms and reveal functional variety etc., at the transcriptional level, which consummated the genome database offers a scientific basis for molecular breeding [16, 18].

Castor oil is called a “renewable green oil”, with an annual global production as high as 500,000 tons. Developing castor oil resources is one of the most important technical ways to reduce oil dependence, maintain the ecological balance and can enhance rapid economic growth [19]. The main component of castor oil is triglycerides of higher fatty acids. Eight different fatty acids found in castor oil, among, ricinoleic (66%), oleic (8%), palmitic (8%) and linoleic acid (6%) were the major components [19]. The content of unsaturated fatty acids was as high as 88%, which gives castor oil good prospects for development and application [19].

The biosynthetic pathway of castor oil had earlier discovered by Lin et al. [19]. Oleoyl-ACP is first synthesized in plastids, then hydrolyzed to free oleic acids by oleoyl-ACP thioesterase (AAT), and finally converted by acyl-CoA synthase (ACS) to form oleoyl-CoA [20]. Subsequently, oleoyl-CoA is exported into the acyl pool and used as the raw material for the synthesis of ricinolate. Oleoyl-CoA enters the endoplasmic reticulum and the acyl editing pathway, in which lysophosphatidylcholine acyltransferase (LPCAT) transfers the oleoyl group of oleoyl-CoA to the sn-2 site of phosphatidylcholine (PC) as the substrate of oleoyl-12-hydroxylase (FAH12), which catalyzes the conversion of 2-oleoyl-PC to 2-ricinoleoyl-PC. Phospholipase A2 (PLA2) releases ricinoleate and lysophosphatidylcholine (LPC) from 2-ricinoleoyl-PC simultaneously. LPC can form sn-2-oleoyl-PC again as the substrate of FAH12, in a reaction catalyzed by LPCAT. Ricinoleate is converted into ricinoleoyl-CoA by long-chain lipoyl-CoA synthetase (LACS), which acts as an acyl donor that enters the acyl-CoA-dependent pathway, and finally TAG is formed by acylation reactions. The glycerol-3-phosphate acyltransferase (GPAT) first forms ricinoleyl-*lyso*PA. Lysophosphatidic acid acyltransferase (LPAAT), the second acyltransferase enzyme catalyzes the acylation of the LPA site to produce phosphatidic acid (PA). PA is phosphorylated by phosphatidic acid phosphatase (PAP) to yield diacylglycerol (DAG). Diacylglycerol acyltransferase (DGAT), the third acyltransferase, catalyzes the acylation of the sn-3 site of DAG to form TAG. The fatty acid specificity of these acyltransferases determines the composition of fatty acids in the TAGs. The key enzymatic steps are catalyzed by acyl-ACP thioesterase, lysophosphatidylcholine acyltransferase, oleoyl-12-hydroxylase, phospholipase A2, diacylglycerol acyltransferase and a ratio of phosphatidylcholine:diacylglycerol acyltransferase. Furthermore, Lin at al. (2007) reported that phospholipase C2 (PLC2) catalyzes the conversion of 2-oleoyl-PC into 1-acyl-2-oleoyl-sn-glycerol. It may prevent the incorporation of hydroxy fatty acids into TAG and increase the content of ricinoleic acid in the seeds [19]. Apart from acyl-CoA-dependent pathway, acyl-CoA-independent pathway can also produce TAGs.

This study firstly unveils the full-length transcriptome sequences from *R. communis* obtained through the PacBio SMRT technique compare with Illumina RNA sequencing to generate a more complete transcriptome, and further to detect AS-variants, fusion genes, lncRNAs and APA-sites. Illumina RNA-seq was also used in this study to improve the PacBio SMRT transcript isoforms through short-read error correction and comparison of the differences between transcripts from the two platforms. In addition, Illumina RNA-seq transcriptome analysis of the immature seeds of two *R. communis* lines with extremely different castor oil contents, 349 and 1115, was used to identify the genes related to ricinoleic acid biosynthesis. Our results have the potential to improve *R. communis* genome existing model, contribute towards understanding of the complexity of the *R. communis* genome, and serve as reference sequences for differential expression analysis in the future. The annotation of DEGs between two varieties with extremely different levels of oil content will help further understanding of the detailed mechanism of ricinoleic acid biosynthesis in *R. communis.*

## Results

### PacBio SMRT sequencing

**Fig. 1:**
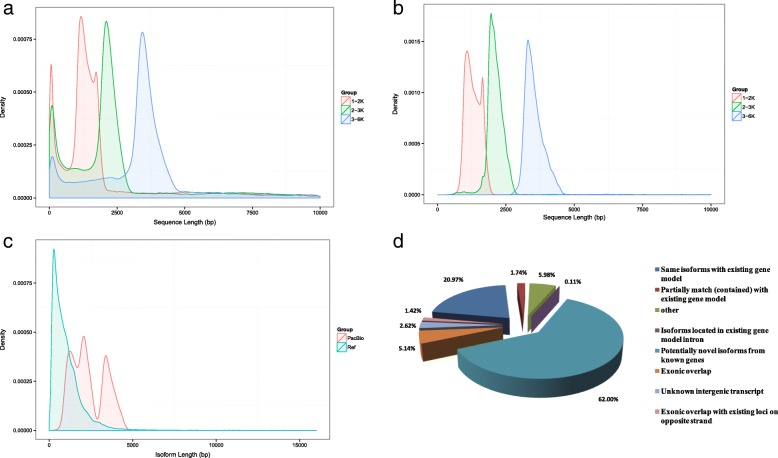
Library construction of PacBio SMRT sequencing and isoform comparison between the *Ricinus communis* genome and full-length transcriptome. **a** Quality inspection of reads of inserts (ROI) in three libraries (1–2 k, 2-3 k and 3-6 k). **b** Quality inspection of full-length non-chimeric (FLNC) reads in three libraries (1–2 k, 2-3 k and 3-6 k). **c** Isoform length comparison between the reference genome and PacBio long-reads data. **d** Comparison of isoforms sequences between the *Ricinus communis* genome and full-length transcriptome

**Fig. 2:**
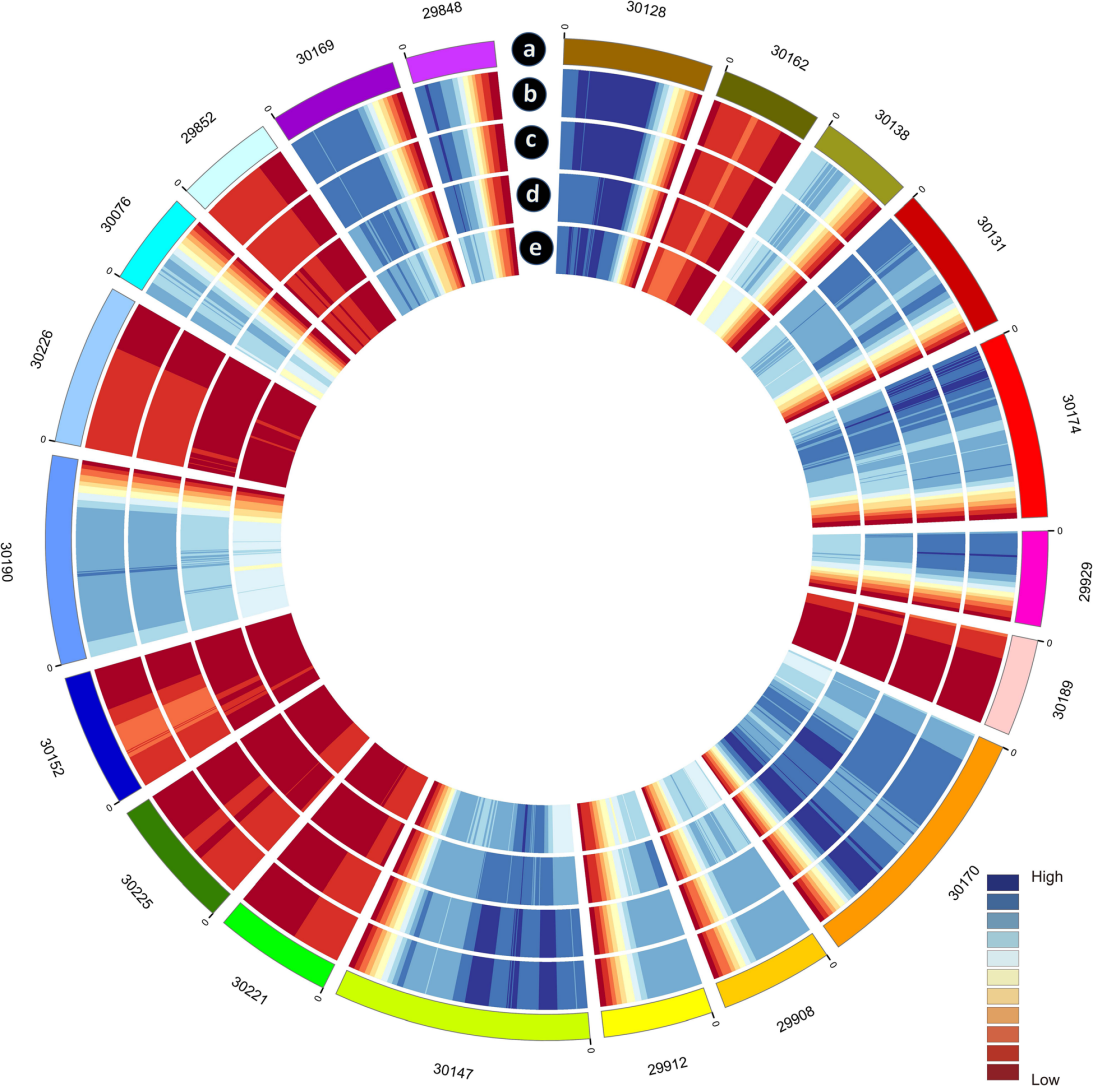
CIRCOS visualization of gene and transcript density compared PacBio SMRT sequences with *Ricinus communis* reference genome for 20 top lengths of scaffolds. **a** Twenty longest scaffolds schematic. **b** Heat map of gene density distribution of PacBio SMRT sequences. Gene density was calculated in a 1-Mb sliding window at 20 kb intervals. **c** Heat map of transcripts density distribution of PacBio SMRT sequences. Gene density was calculated in a 1-Mb sliding window at 20 kb intervals. **d** Heat map of gene density distribution of the reference genome. Gene density was calculated in a 1-Mb sliding window at 20 kb intervals. **e** Heat map of transcripts density distribution of the reference genome. Gene density was calculated in a 1-Mb sliding window at 20 kb intervals

To better identify full-length splice variants, novel genes, APA-sites etc., PacBio Iso-Seq platform was applied to sequence the transcriptome of *R. communis* on the basis of the Illumina HiSeqTM 2500 sequencing platform (Illumina Inc., San Diego, CA, US). A total of 3 cells (1–2 kb, 2-3 kb and 3-6 kb) were constructed to eliminate bias of the instrument for short fragments (Fig. 1a, b). A dataset with 9.42 Gb of clean reads was obtained after filtering using SMRTLink (4.0). The mean length of clean reads in the three libraries was between 1698 and 4132 bp (Additional file 1: Table S1). A total of 242,942 reads of insert (ROI) were screened with full passes ≥0 and accuracy of sequence was set at ≥0.75. The mean length of the ROI in the three libraries was between 2088 and 4013 bp (Additional file 1: Table S2, Additional file 2: Figure S1). The number of full-length reads, which contained poly-A, 5′ and 3′ primers, was 111,507 (45.90% of total ROI). There were 110,469 full-length non-chimeric reads with a mean length of 2201 bp (Additional file 1: Table S3). In total, 50,636 high-quality isoforms were identified (Additional file 1: Table S4). The isoform lengths of the PacBio SMRT sequences were longer than those of the reference genome (Fig. 1c), which indicated that more full-length and novel isoforms were identified based on PacBio SMRT sequencing. Mapping the results based on the reference genome revealed that a total of 20.97% of the isoforms detected by PacBio SMRT sequencing were the same with the corresponding genomic annotations, 1.74% of all transcripts were partially mapped to the genome, and 62.00% potentially were novel isoforms (Fig. 1d). The 20 longest scaffolds (Fig. 2a) of *R. communis* genome were selected to compare the isoforms density. Thus, the result showed that PacBio SMRT sequencing data had higher gene (Fig. 2b, d) and transcript density (Fig. 2c, e) than the *R. communis* reference genome. Consequently, the *R. communis* genome was enriched with the PacBio SMRT results and used for further analysis.

### Detection of alternative splicing events and novel genes

**Fig. 3:**
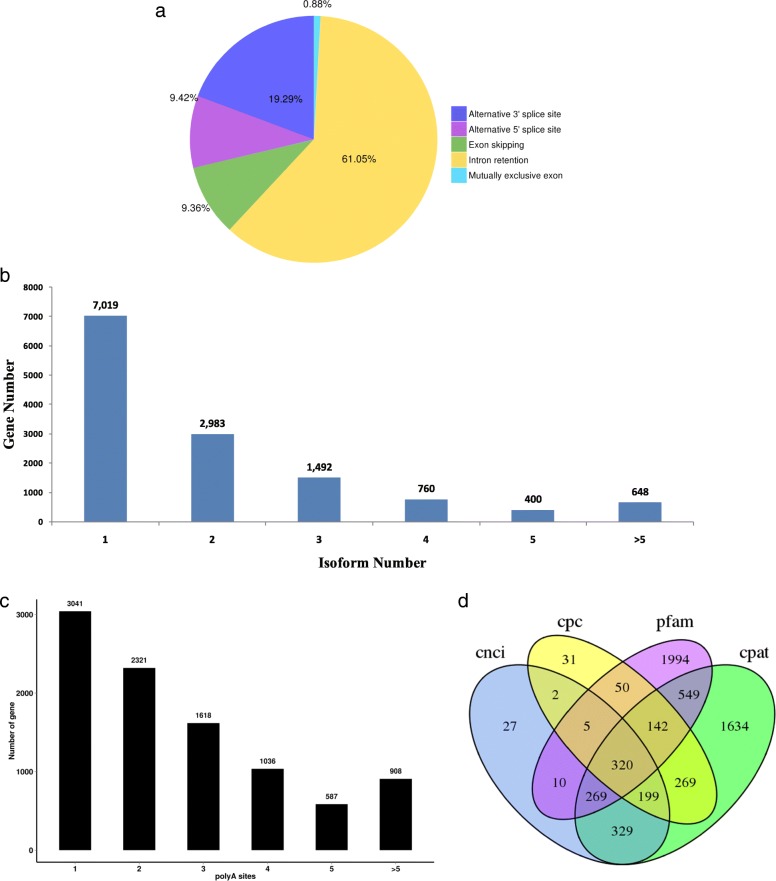
Identification of lncRNAs, alternative splicing events, isoform numbers and alternative polyadenylation (APA) based on transcriptome technologies. **a** Number and categories of alternative splicing events based on the PacBio platform. **b** Number and categories of isoforms based on the PacBio platform. **c** Number and categories of APA based on the PacBio platform. **d** Number of long non-coding RNAs analyzed by CNCI, CPC, PFAM and CPAT based on the PacBio platform

Pre-mRNA, a precursor in eukaryotic gene transcription, has multiple splicing patterns. Five main AS events (alternative 3′ splice sites, alternative 5′ splice sites, retained introns, skipped exons and mutually exclusive exons) were identified. A total of 11,285 AS events were found in *R. communis* based on the PacBio SMRT reads. The number of three kinds of events, alternative 3′ splice sites (2177), alternative 5′ splice sites (1063) and retained introns (6890), were much higher than those of skipped exons (1056) and mutually exclusive exons (99). Among these, a new gene *PB.2740* had the largest number of splice variants (23 transcripts) according to the PacBio SMRT sequencing data. There were no significant differences among other plants in the distribution of AS events, with the majority of AS events being retained introns (Fig. 3a). To some extent, alternative splicing allows the same gene to produce multiple isoforms. Isoform numbers from PacBio SMRT reads are summarized in Fig. 3b. As the reference genome annotations are not accurate, it is necessary to optimize the structure of the original annotated genes. If mapped reads support in areas outside the original gene boundaries, we extended the gene’s untranslated region (UTR) upstream and downstream to correct the gene boundary. The gene structure optimization results are shown in Additional file 1: Table S5. The Prediction of novel genes may enrich the genome information of *R. communis* and offer a new direction to further study ricinoleic acid biosynthesis. A total of 21,448 novel genes were identified using PacBio SMRT sequencing approach and 98.48% (21,122) were annotated according to information from the Cluster of Orthologous Groups (COG) of proteins [21] (8991), the gene ontology (GO) [22] (15,841), the Kyoto Encyclopedia of Genes and Genomes (KEGG) [23] (9267), the protein family (Pfam) [24] (16,822), the manually annotated and reviewed protein sequence database (SwissProt) [25] (15,188) and Non-redundant protein (NR) [26] (21,112) databases (Additional file 1: Table S6).

### Identification of fusion genes, alternative polyadenylation, transcription factor and LncRNAs

**Fig. 4:**
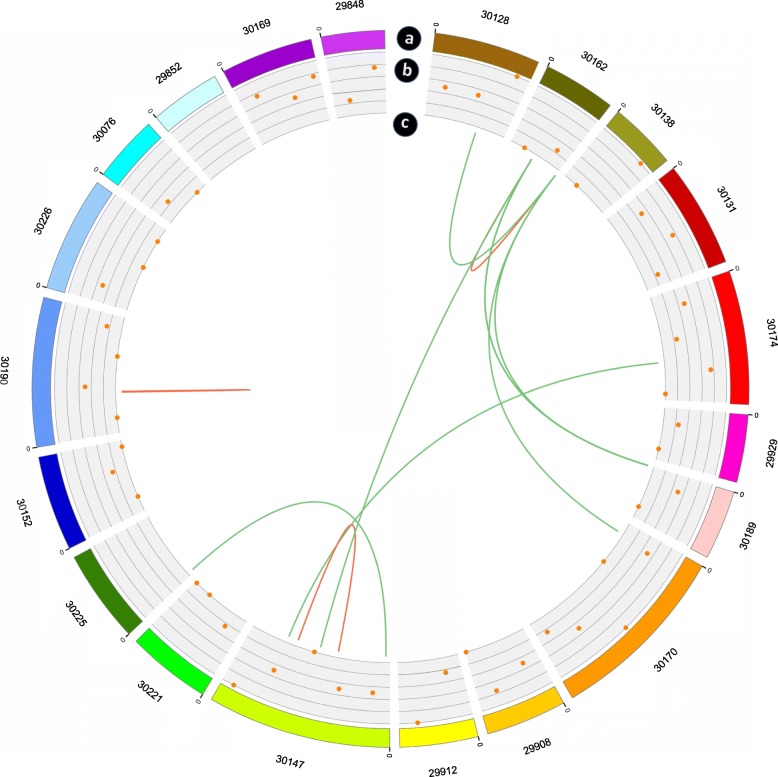
CIRCOS visualization of lncRNA density and linkage of fusion transcripts for 20 top lengths of scaffolds. **a** Twenty longest scaffolds schematic. **b** LncRNA density, in 1 Mb bins on each chromosome. **c** Linkage of fusion transcripts: red, intra-chromosomal; green, inter-chromosomal

A fusion gene is a chimeric gene composed of two or more separate genes [5]. A total of 250 fusion genes were identified in the SMRT-seq library. The detailed information of 250 fusion genes was supplied in Additional file 1: Table S7. Our results revealed that all fusion events were comprised of two genes, which was consistent with previous reports that fusion events mostly involve two genes [27]. In eukaryotes, APA of pre-mRNA may contribute to transcriptome diversity, genome coding capacity and gene regulation. A total of 9511 APA genes were identified using the TAPIS pipeline. Among these, 3041 genes (36.15%) with a single poly-A site were detected, and the number of genes with two or more poly-A sites was 5562. For 908 genes, five or more poly-A sites were detected (Fig. 3c). Transcription factors (TFs) account for a large part of the plant genome and play a vital part in gene regulation. In our study, a total of 1356 genes were annotated as encoding TFs from 69 families according to SMRT-seq. Using our data, we identified novel genes from 58 families, increasing the number of TF isoforms to 2335 (Additional file 1: Table S8). Because lncRNAs do not encode proteins, the transcript can be screened to determine whether it has potential of coding ability. If one has no potential coding ability, it would be categorized as a bona-fide lncRNA. Four coding potential analysis methods were used to predict the lncRNAs among the novel transcripts. These methods included Coding Potential Calculator (CPC) [28], Coding-Non-Coding Index (CNCI) [29], Pfam [24] and Coding Potential Assessment Tool (CPAT) [30]. A total of 320 lncRNAs were found among all the datasets, including CPC (1018), CNCI (1161), CPAT (3711) and Pfam (3339) (Fig. 3d). Of the 320 lncRNAs, 154 lincRNAs, 57 antisense-lncRNAs, 15 intronic-lncRNAs and 56 sense-lncRNAs were identified. Moreover, the 20 longest scaffolds of *R. communis* genome were selected to study the fusion genes and lncRNAs identified by SMRT-seq. Mapping lncRNAs to the 20 longest scaffolds (Fig. 4a) revealed that they have similar distribution to that of protein-coding genes, which are enriched outside of pericentromeric regions (Fig. 4b). Ten fusion events of the 20 longest scaffolds by SMRT-seq were more likely to occur at inter-chromosomally (7) than intra-chromosomally (3) (Fig. 4c).

### Illumina RNA sequencing

To analyze the ricinoleic acid biosynthesis mechanism, we selected the two cultivars 349 and 1115 with extremely different castor oil content, and analyzed their different tissues at three-time points with triplicate condition for Illumina RNA-seq, resulting in a total of 18 libraries. Correlation analysis results of three replications of 18 samples revealed Pearson’s correlation coefficients between 0.922 and 0.999. Notably, all these values were greater than the 0.92 recommended under ideal experimental conditions (Additional file 2: Figure S2). A total of 121.50 Gb of clean data (Q30 > 90.14%) was generated after filtering the original data, the GC contents were between 42.01 and 46.01% (Additional file 1: Table S9). The error rate of all clean data per sample was controlled below 0.05%. The amount of paired-end reads among the clean reads ranged from 20,165,714 to 25,302,539 among the 18 samples, of which between 81.72 and 88.56% were successfully mapped to the reference genome. Of these mapped data, the percentage of samples that uniquely mapped to the genome was between 70.31 and 87.53% (Additional file 1: Table S10).

### Analysis of the DEGs

**Fig. 5:**
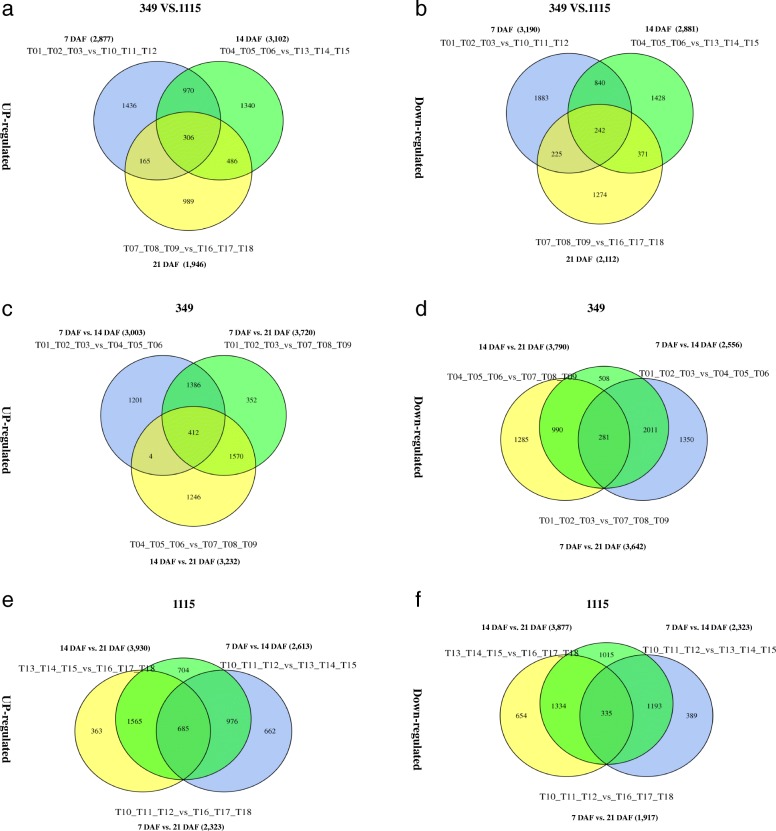
Venn diagrams of differentially expressed genes (DEGs) between *R. communis* strains 349 and 1115 at three time points. **a** Number of up-regulated DEGs among 7 DAF, 14 DAF and 21 DAF (349 vs. 1115) (**b**) Number of down-regulated DEGs among 7 DAF, 14 DAF and 21 DAF. **c** Number of up-regulated genes among 7 DAF vs. 14 DAF, 7 DAF vs. 21 DAF and 14 DAF vs. 21 DAF (349). **d** Number of down-regulated genes among 7 DAF vs. 14 DAF, 7 DAF vs. 21 DAF and 14 DAF vs. 21 DAF (349). **e** Number of up-regulated genes among 7 DAF vs. 14 DAF, 7 DAF vs. 21 DAF and 14 DAF vs. 21 DAF (1115). **f** Number of down-regulated genes among 7 DAF vs. 14 DAF, 7 DAF vs. 21 DAF and 14 DAF vs. 21 DAF (1115)

DESeq software [31] was used to analyze biological triplicate samples obtained from DEG screening. In 349 and 1115 cultivars, a total of 6067 (2877 up and 3190 down) DEGs were identified at 7 days after flowering (DAF), while the up- and down-regulated DEGs at 14 DAF were 3102 and 2881. At 21 DAF, these were 1946 and 2112, respectively. Among all genes, 306 up- and 242 down-regulated DEGs were expressed in both genotypes at all three stages. Furthermore, 1810 (970 up and 840 down) DEGs were expressed in both at 7 DAF and 14 DAF, and 390 (165 up and 225 down) DEGs were expressed in both strains at 7 DAF and 21 DAF. The number of stage-specifically expressed DEGs was 1436 up and 1883 down (7 DAF), 1340 up and 1428 down (14 DAF), and 989 up and 1274 down (21 DAF) (Fig. 5a-b). For genotype 349, 3003 up- and 2556 down-regulated DEGs were obtained between 7 DAF and 14 DAF, while the up- and down-regulated DEGs between 7 DAF and 21 DAF were 3720 and 3642. Between 14 DAF and 21 DAF, these were 3232 and 3790, respectively. A total of 412 up- and 281 down-regulated DEGs were identified common among 7 DAF, 14 DAF and 21 DAF (Fig. 5c-d). Similarly, in genotype 1115, 2613 up- and 2323 down-regulated DEGs were obtained between 7 DAF and 14 DAF, while the up- and down-regulated DEGs between 7 DAF and 21 DAF were 2323 and 1917. Between 14 DAF and 21 DAF were 3930 and 3877, respectively. A total of 685 up- and 335 down-regulated DEGs were identified among 7 DAF, 14 DAF and 21 DAF (Fig. 5e-f).

### Annotation analysis of DEGs

To better understand the function of the DEGs and further reveal the mechanism regulating ricinoleic acid biosynthesis, all DEGs were obtained in 349 and 1115 were blast with GO, COG, Pfam, KEGG, SwissProt and NR databases using Basic Local Alignment Search Tool (BLAST) software [32]. All DEGs at three time points were successfully annotated using the above mentioned seven databases (Table 1).

**Table 1:** Summary of annotated differentially expressed genes between *R. communis* strains 349 and 1115 at three time points (7 DAF, 14 DAF and 21 DAF)

DEG Set	Total	COG	eggNOG	NR	Pfam	Swiss-Prot	GO	KEGG
349 vs. 1115 at 7 DAF	6067	2391	5798	5940	4985	4449	4527	2061
349 vs. 1115 at 14 DAF	5983	2393	5719	5862	4950	4393	4460	1969
349 vs. 1115 at 21 DAF	4058	1610	3845	3963	3307	2968	3037	1359

For the GO classification analysis, 4527, 4460 and 3037 DEGs at three time points were respectively assigned to the three main GO functional categories and then divided into 51, 50, and 47 sub-categories (Additional file 2: Figure S3). In biological process, the dominant terms at all three time points (7, 14 and 21 DAF) were “metabolic process”, “cellular process”, and “single-organism process”. However, “locomotion” and “biological phase” could not be detected at 21 DAF. For cellular components, “cell part”, “cell”, “organelle”, “membrane”, “membrane part”, and “macromolecular complex”, had accounted for the majority. The “extracellular matrix part” was not identified at 21 DAF. Regarding molecular function, the top three terms at the three time points were “binding”, “catalytic activity” and “transporter activity”. The term “metallochaperone activity” was only found at 7 DAF.

The transcriptome profile of metabolic pathways of immature seeds of *R. communis* is valuable for understanding the physiological processes of ricinoleic acid biosynthesis. To evaluate the metabolic differences between the two lines at three different time points, an analysis using the KEGG database on biological pathways showed that a total of 2061, 1969 and 1359 DEGs were assigned to 50 pathways (Additional file 2: Figure S4). Three pathways (“Carbon metabolism”, “Biosynthesis of amino acids” and “Ribosome”) were the most abundant at 7 DAF and 21 DAF, while “Carbon metabolism”, “Biosynthesis of amino acids” and “Starch and sucrose metabolism” were the top three pathways at 14 DAF. Pathway enrichment analysis of DEGs is a way to indicate whether there are significant differences in a certain pathway, and the hypergeometric test [33] was used to find the pathway that was significantly enriched in the DEGs compared with the whole-genome background. The first 20 pathways with significant *p*-values are presented in Additional file 2: Figure S4. The statistics of pathway enrichment of the three time points showed that the significant differences of DEGs in pathways at 7 DAF were much higher than at 14 and 21 DAF.

In the COG database, the dominant terms at 7 DAF in “General function prediction only”, were “Transcription, Replication” and “Recombination and repair”. Similarly, the top three classes at 14 DAF were “Transcription, Replication”, and “Signal transduction mechanisms”, while three pathways such as “Signal transduction mechanisms” and “Replication, recombination and repair” were the most abundant at 21 DAF (Additional file 2: Figure S5).

### Identification of candidate genes related to ricinoleic acid biosynthesis and quantitative real-time reverse transcription PCR confirmation

Ricinoleate (R), a hydroxy fatty acid (FA), has many industrial uses, including the manufacture of aviation lubricants, plastics, paints, and cosmetics. Castor oil is the only commercial source of ricinoleate. The biosynthetic pathway of triricinolein (RRR) and (12-ricinoleoylricinoleoyl) diricinoleoylglycerol (RRRR) in *R. communis* has been established [19], and the key enzymatic steps driving the conversion of ricinoleate into RRR and RRRR have been identified [19]. Several key enzymes were found to be acyl-ACP thioesterases [34], oleate 12-hydroxylase [35], diacylglycerol acyltransferase [36], phosphatidylcholine:diacylglycerol acyltransferase [37], phospholipase C2 [19], phospholipase A2 [38], lysophosphatidylcholine acyltransferase [39] and etc.. By analyzing annotated information of all DEGs between two genotypes were 349 and 1115 at the three-time points, a total of 14, 18 and 11 DEGs related to ricinoleic acid biosynthesis were identified at 7, 14 and 21 DAF, respectively. Several DEGs related to the key enzymes for ricinoleic acid biosynthesis at the three-time points were selected for quantitative RT-PCR (qRT-PCR) analysis to validate the Illumina RNA sequencing data. The results showed a consistent expression trend between RNA sequencing and qRT-PCR.

**Fig. 6:**
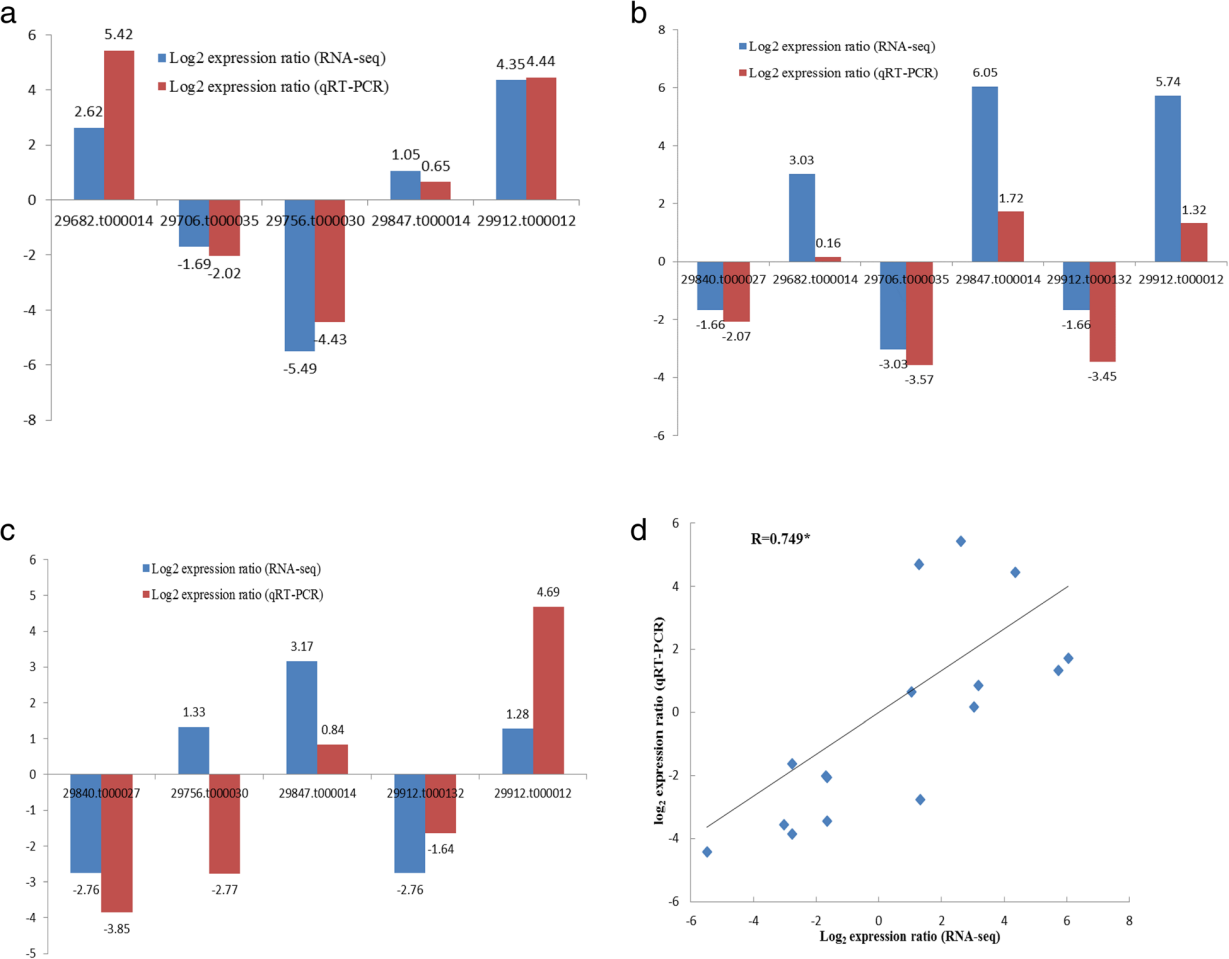
QRT-PCR validation of several DEGs related to the key enzymes for ricinoleic acid biosynthesis at three time points. **a** The RNA-Seq log_2_ values (expression ratios of 349-RPKM/1115-RPKM) and the qRT-PCR log_2_ values (expression ratios of 349/1115) of five important DEGs between strains 349 and 1115 at 7 DAF. **b** The RNA-Seq log_2_ values (expression ratios of 349-RPKM/1115-RPKM) and the qRT-PCR log_2_ values (expression ratios of 349/1115) of six important DEGs between strains 349 and 1115 at 14 DAF. **c** The RNA-Seq log_2_ values (expression ratios of 349-RPKM/1115-RPKM) and the qRT-PCR log_2_ values (expression ratios of 349/1115) of five important DEGs between strains 349 and 1115 at 21 DAF. **d** Correlation analysis of the DEG expression ratios obtained from the qRT-PCR and RNA-seq data of 16 DEGs (*p*-value < 0.05)

At 7 DAF, 2 DEGs *29,848.t000233* (down-regulated) and *30,217.t000013* (up-regulated) had encoded acyl-ACP thioesterase enzyme. Four DEGs, *30,147.t000759* (down-regulated), *30,142.t000002* (up-regulated), *29,847.t000014* (up-regulated) and *29,801.t000111* (up-regulated) related to phospholipase C were identified using the KEGG, NR and SwissProt databases. Only one DEG *30170.t000326* (down-regulated) encoding an enzyme with phospholipase A2 activity was annotated. Three DEGs *30,131.t000161* (down-regulated), *29,912.t000099* (down-regulated) and *29,682.t000014* (up-regulated) were found to encode diacylglycerol acyltransferases. Two DEGs *29,991.t000008* (up-regulated) and *29,706.t000035* (down-regulated) were found to encode the ratio of phosphatidylcholine:diacylglycerol acyltransferase (Table 2). The expression levels of 5 genes (*29,682.t000014*, *29,706.t000035*, *29,756.t000030*, *29,847.t000014* and *29,912.t000012*) at 7 DAF were confirmed by qRT-PCR analysis to validate the Illumina RNA-seq data (Fig. 6a).

**Table 2:** List of DEGs encoding key enzymes of ricinoleic acid biosynthesis at 7 DAF

Stages	Key enzyme	Gene ID	Databases	Annotation	Up/Down
7 DAF	AAT	*29,848.t000233*	KEGG	Fatty acyl-ACP thioesterase B (K10781)	Down
			Pfam	Acyl-ACP thioesterase	
		*30,217.t000013*	KEGG	Fatty acyl-ACP thioesterase B (K10782)	Up
			Pfam	Acyl-ACP thioesterase	
		*29,756.t000030*	GO	Phospholipase C activity (GO:0004629)	Down
			KEGG	Phospholipase C (K01114)	
	PLC	*30,147.t000759*	KEGG	Phospholipase C (K01114)	Down
			NR	Phospholipase C 4 precursor	
		*30,142.t000002*	KEGG	Phospholipase C (K01114)	Up
			Swiss-Prot	Phospholipase C2 (Precursor)	
		*29,847.t000014*	NR	Phospholipase C	Up
		*29,801.t000111*	NR	Phospholipase C	Up
	PLA_2_	*30,170.t000326*	GO	Phospholipase A2 activity (GO:0004623)	Down
	DGAT	*30,131.t000161*	Pfam	Diacylglycerol acyltransferase	Down
		*29,912.t000099*	KEGG	Type 1 diacylglycerol acyltransferase (K11155)	Down
			NR	Type 1 diacylglycerol acyltransferase	
		*29,682.t000014*	KEGG	Type 2 diacylglycerol acyltransferase (K14457)	Up
			Pfam	Diacylglycerol acyltransferase	
	PDAT	*29,991.t000008*	KEGG	Phospholipid:diacylglycerol acyltransferase (K00679)	Up
			NR	Phosphatidylcholine: Diacylglycerol Acyltransferase	
		*29,706.t000035*	KEGG	Phospholipid:diacylglycerol acyltransferase (K00679)	Down
			NR	Phosphatidylcholine: Diacylglycerol Acyltransferase	
		*29,912.t000012*	Swiss-Prot	Phospholipid:diacylglycerol acyltransferase	Up

Compared 14 DAF with 7 DAF, the DEGs of key enzymes related to ricinoleic acid biosynthesis showed some interesting differences. For instance, *30,147.t000739* (down-regulated) was related to acyl-ACP thioesterase, *30,147.t000759* (up-regulated) and *29,847.t000014* (up-regulated) related to phospholipase C and *30,142.t000005* (down-regulated), *28,470.t000001* (down-regulated), *29,912.t000132* (down-regulated) and *29,840.t000027* (up-regulated) were related to phospholipase A2. Moreover, *30,128.t000117* (down-regulated) encoding diacylglycerol acyltransferase was identified at 14 DAF. For DEGs related to phospholipid:diacylglycerol acyltransferase, there were no differences between 7 and 14 DAF. Only one DEG *30170.t000414* (down-regulated) encoding lysophosphatidyl acyltransferase was found at 14 DAF, while no DEG was identified at 7 DAF (Table 3). The expression levels of 6 genes (*29,840.t000027*, *29,682.t000014*, *29,706.t000035*, *29,847.t000014*, *29,912.t000132* and *29,912.t000012*) at 14 DAF were also confirmed by qRT-PCR analysis (Fig. 6b).

**Table 3:** List of DEGs encoding key enzymes of ricinoleic acid biosynthesis at 14 DAF

Stages	Key enzyme	Gene ID	Databases	Annotation	Up/Down
14 DAF	AAT	*29,848.t000233*	KEGG	Fatty acyl-ACP thioesterase B (K10781)	Down
			Pfam	Acyl-ACP thioesterase	
		*30,217.t000013*	KEGG	Fatty acyl-ACP thioesterase B (K10782)	Up
			Pfam	Acyl-ACP thioesterase	
		*30,147.t000739*	KEGG	Fatty acyl-ACP thioesterase B (K10781)	Down
			Pfam	Acyl-ACP thioesterase	
	PLC	*30,147.t000759*	KEGG	Phospholipase C (K01114)	Up
			NR	Phospholipase C 4 precursor	
		*29,847.t000014*	NR	Phospholipase C, putative	Up
	PLA_2_	*30,170.t000326*	GO	Phospholipase A2 activity (GO:0004623)	Down
		*30,142.t000005*	GO	Phospholipase A2 activity (GO:0004623)	Down
		*28,470.t000001*	KEGG	Phospholipase A2 (K14674)	Down
		*29,912.t000132*	Pfam	Phospholipase A2	Down
			Swiss-Prot	Probable phospholipase A2 homolog 1	
		*29,840.t000027*	Pfam	Phospholipase A2	Up
			Swiss-Prot	Phospholipase A2-alpha (Precursor)	
	DAGT	*30,131.t000161*	Pfam	Diacylglycerol acyltransferase	Down
		*29,912.t000099*	KEGG	Type 1 diacylglycerol acyltransferase (K11155)	Down
			NR	Type 1 diacylglycerol acyltransferase	
		*29,682.t000014*	KEGG	Type 2 diacylglycerol acyltransferase (K14457)	Up
			Pfam	Diacylglycerol acyltransferase	
		*30,128.t000117*	Pfam	Diacylglycerol acyltransferase	Down
	PDAT	*29,991.t000008*	KEGG	Phospholipid:diacylglycerol acyltransferase (K00679)	Up
			NR	Phosphatidylcholine: Diacylglycerol Acyltransferase	
		*29,706.t000035*	KEGG	Phospholipid:diacylglycerol acyltransferase (K00679)	Down
			NR	Phosphatidylcholine: Diacylglycerol Acyltransferase	
		*29,912.t000012*	Swiss-Prot	Phospholipid: diacylglycerol acyltransferase	Up
	LPCAT	*30,170.t000414*	KEGG	Lysophosphatidylcholine acyltransferase (K13510)	Down
			Swiss-Prot	Lysophospholipid acyltransferase	

**Fig. 7:**
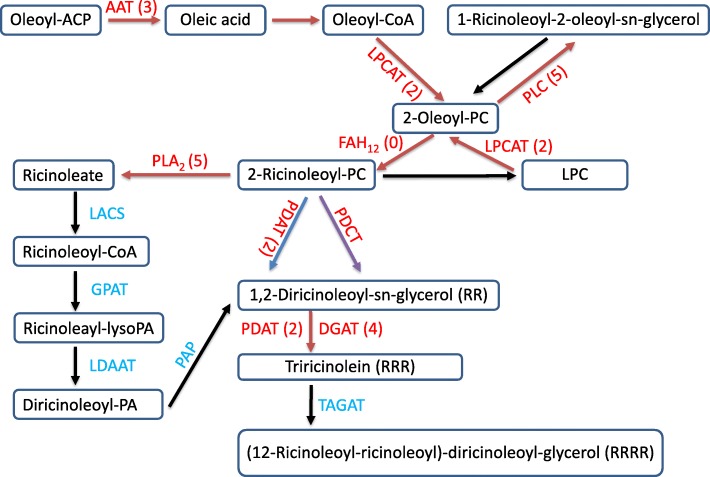
Illustration of DEGs encoding the key enzymes of the ricinoleic acid biosynthetic pathway

At 21 DAF, only one DEG, *30147.t000739* (up-regulated) related to acyl-ACP thioesterase was identified. Here, we had found a novel DEG *29756.t000030* (up-regulated) encoding phospholipase C. However, the transcript of *30,147.t000759* disappeared at 21 DAF, while *30,142.t000002* and *29,801.t000111* expressed only at 21 DAF. Compared to 14 DAF, only two DEGs, *30,170.t000326* and *29,840.t000027* were found to encode phospholipase A2. No DEGs related to diacylglycerol acyltransferase were found at 21 DAF. Only one DEG related to phospholipid:diacylglycerol acyltransferase *29,991.t000008* was identified at 21 DAF. By comparison, one more DEG, *30174.t000334* (down-regulated), encoding lysophosphatidyl acyltransferase was found at 21 DAF (Table 4). The expressional levels of 4 genes (*29,840.t000027*, *29,847.t000014*, *29,912.t000132* and *29,912.t000012*) at 21 DAF were also confirmed by qPCR analysis, but not that of *29,756.t000030*, whose expressional level differed from qPCR data (Fig. 6c). Moreover, a correlation analysis (Fig. 6d) of expression levels of 7 selected candidate genes related to ricinoleic acid biosynthesis at the three-time points (7, 14 and 21 DAF) showed a significant correlation (correlation coefficient R = 0.749, *p*-value < 0.05) between qRT-PCR and Illumina RNA-seq, suggesting that the data generated in the RNA-seq assay of this study are of sufficiently high quality for investigating the differential expression of genes between *R. communis* two strains 349 and 1115. To better understand the complex ricinoleic acid biosynthesis processes, we constructed a putative model based on pivotal data at the transcriptional level (Fig. 7).

**Table 4:** List of DEGs enconding key enzymes of ricinoleic acid biosynthesis at 21 DAF

Stages	Key enzyme	Gene ID	Databases	Annotation	Up/Down
21 DAF	AAT	*30,147.t000739*	KEGG	Fatty acyl-ACP thioesterase B (K10781)	Up
			Pfam	Acyl-ACP thioesterase	
	PLC	*30,142.t000002*	KEGG	Phospholipase C (K01114)	Up
			Swiss-Prot	Phospholipase C2 (Precursor)	
		*29,847.t000014*	NR	Phospholipase C, putative	Up
		*29,801.t000111*	NR	Phospholipase C, putative	Down
		*29,756.t000030*	GO	Phospholipase C activity (GO:0004629)	Up
			NR	Phospholipase C, putative	
	PLA_2_	*30,170.t000326*	GO	Phospholipase A2 activity (GO:0004623)	Down
		*29,840.t000027*	Pfam	Phospholipase A2	Up
			Swiss-Prot	Phospholipase A2-alpha (Precursor)	
	PDAT	*29,991.t000008*	KEGG	Phospholipid:diacylglycerol acyltransferase (K00679)	Down
			NR	Phosphatidylcholine: Diacylglycerol Acyltransferase	
		*29,912.t000012*	Swiss-Prot	Phospholipid:diacylglycerol acyltransferase	Up
	LPCAT	*30,170.t000414*	KEGG	Lysophosphatidylcholine acyltransferase (K13510)	Down
			Swiss-Prot	Lysophospholipid acyltransferase	
		*30,174.t000334*	KEGG	Lysophosphatidylcholine acyltransferase (K13510)	Down
			Swiss-Prot	Lysophospholipid acyltransferase

## Discussion

The ricinoleic acid of castor oil has a great commercial value. Understanding the process of TAG assembly and the accumulation of nearly 90% of ricinoleic acid in castor oil are provided the basis of successful research on transgenic crops [19]. Similarly, the study on the basic metabolic pathways of related lipids is also conducive to the production of other highly economically valuable fatty acids through genetic engineering. It has been reported that the accumulation of ricinoleate in *R. communis* was the result of substrate-specific regulation of related enzymes [19]. Several key enzymes have been identified, including AAT, FAH12, DGAT, PDAT, PLA2, PLC2 and LPCAT, which play vital roles in the fatty acid metabolism of developing castor beans. Though genetic modification of related genes, it is possible to alter the composition of fatty acids, allowing genetically modified plants to produce ricin-free seed to increases the use of plant fat as a renewable low-carbon resource. In this study, two genotypes (*R. communis* strains 349 and 1115) with contrasting castor oil content were selected to better understand the ricinoleic acid biosynthesis through PacBio SMRT and Illumina RNA sequencing at three-time scales (7, 14 and 21 DAF). After transcriptome analysis, some candidate genes were identified putatively play key roles in RRR synthesis. The accumulation of lipids involves several biosynthetic and catabolic pathways, and it is impossible to significantly increase lipid content by simply expressing a specific gene [39]. Nevertheless, the cumulative expression of the relevant genes in transgenic plants may lead to higher levels of castor oil in the future.

### Characteristics of the genome sequence enriched with PacBio SMRT reads and analysis of LncRNAs, AS events and fusion genes

In our study, the percentage mapped to the known next-generation genome had reached to 85.94%. However, a total of 22.71% of isoforms detected in SMRT-seq were totally or partially mapped to the genome. Almost 62% of isoforms were potentially novel transcripts of known genes means that the information content of the enriched genome was larger than known genome. Moreover, by analyzing the 20 longest scaffolds, it was known PacBio SMRT-seq data had higher gene and transcript density than the *R. communis* reference genome. It indicates that the new transcriptome data has a great potential to improve the current version of *R. communis* annotation. A large number of new genes and exons were identified and made available for researching genes function and structure, which could provide new insights for improving castor oil content. LncRNAs are not translated into polypeptides. Nevertheless the expression and transcriptional regulation of genes is significantly affected by them [40, 41]. We firstly identified lncRNAs in *R. communis* during seed development, but their functions remain unknown. Through mapping newly identified lncRNAs to the 20 longest scaffolds of *R. communis* genome, our work solidifies the conclusion that lncRNAs have a similar distribution to that of protein-coding genes and enriched outside of pericentromeric regions which has been proved in maize previously [42]. AS events are one of the key elements driving the diversity of genes and proteins in eukaryotes [43]. Although several AS events were identified in the current study highlighted the differences of castor oil contents between strains 349 and 1115 needs to be investigated further in the future. Consistent with the maize transcriptome by PacBio SMRT-seq [42], we also identified the higher proportion of inter-chromosomal (70%) to intra-chromosomal (30%) fusions from observing the 20 longest scaffolds. Based on these results, our work enormously had improved the existing gene models in *R. communis* and laid the foundation for future research. To insight on the structure and function of lncRNAs, AS events and fusion genes are imperative and helpful to better understand ricinoleic acid biosynthesis at the molecular level.

### Transcription factors

In plants, transcription factors account for a large proportion of all proteins. There are at least 1500 transcription factors (TFs) coding genes in *Arabidopsis thaliana*, accounting for more than 5% of the whole genome [44]. A TF may regulate several genes related to a class of traits, thereby effectively changing the related characteristics of plants [45, 46]. In our study, a total of 1356 TFs from 69 families were found by PacBio SMRT sequencing. For instance, *B3*, *HAP3*, *bZIP*, *AP2*, *MYB* and *ARF* regulate seed development in plants, were also found in our results. Previously, a TF called *WRI1* (*Wrinkled 1*) has been reported to regulate TAG biosynthesis mechanism in mutant *Arabidopsis* seeds, which had 80% lower TAG content than wild type [47]. In Maize, the overexpression of *WRI1* significantly increased the seed oil content by 48%, and heterologous expression of the *BnWRI1* gene in *Arabidopsis thaliana* showed increased in seed oil content by 10–40% suggested that the *WRI1* gene has a high application value for improving the oil content of plant seeds in breeding program [46]. Based on the whole-genome sequence of *R. communis*, Tajima et al. [47] used semi-quantitative PCR and western blotting to identify several genes homologous to *WRI1*, which play an important role in TAG storage during seed development. Moreover, Brown et al. [3] identified several TFs such as *bHLH*, *MYB*, *NAC* etc., play important roles in ricinoleic acid metabolism, these findings were consistent to our study. Unfortunately, there were no *WRI1*-related genes among our results. Studying the gene expression of these above-mentioned TFs will be helpful to reveal the mechanism related to the synthesis of castor oil, with important theoretical and practical significance for further improving the oil content in other crops.

## Conclusions

Two genotypes with contrasting castor oil content (*R. communis* strains 349 and 1115) were used to analyze the molecular mechanism of ricinoleic acid biosynthesis by Illumina RNA-seq and SMRT-seq. Our results significantly improved existed gene models of *R. communis* and a putative model of key genes was built to show the differences between strains 349 and 1115, illustrating the molecular mechanism of castor oil biosynthesis. A total of 22 key genes for ricinoleic acid biosynthesis were identified in the developing seeds of the two *R. communis* lines with extreme differences in oil production at three-time points. All these findings improve our knowledge of ricinoleic acid biosynthesis at the molecular level and provide a solid foundation for further studies and engineering for increasing the level of ricinoleic acid in castor beans.

## Methods

### Plant materials and growth conditions

The two *R. communis* (castor) lines 349 and 1115 with extremely different castor oil contents (43.98 and 67.51% by Gas Chromatography/Mass Spectrometry, respectively) used in this study were obtained from National Infrastructure for Crop Germplasm Resources. Seeds were sown and cultivated in the Yangluo experimental field of the OCRI, CAAS, under standard field conditions, from spring to autumn of 2017. A total of 36 tissues (including fruit, male flower, female flower, leaf, stem and root) of the two castor lines 349 and 1115 at 7, 14 and 21 DAF were collected for PacBio SMRT sequencing. Additionally, 18 immature beans [2 genotypes (349 and 1115) × 3 time points (7, 14 and 21 DAF) × 3 biological replicates] were collected for Illumina RNA sequencing. Each sample from the two lines at different stages was immediately frozen and stored at − 80 °C until further use.

### Total RNA extraction and library construction

Total RNA was isolated using the TRIzol reagent (Invitrogen, Carlsbad, CA, USA) according to the manufacturer’s instructions. DNase I (Promega, Madison, USA) was used to remove the residual DNA from the extracted RNA for 30 min at 37 °C. A NanoDrop 2000 UV-Vis spectrophotometer (NanoDrop, Wilmington, DE, USA) was used to qualify and quantify the extracted RNA, and the samples showed a 260/280 nm ratio between 1.8 and 2.2 and an OD260/230 > 1.0.

For obtaining the complete information of all transcripts, full length transcriptome sequencing was adopted in the present study. The best RNA sample of three replicates was selected from each of the samples and then mixed together as one sample in an equal quantity for PacBio SMRT-seq. The full-length cDNAs were synthesized using the SMRTer PCR cDNA Synthesis Kit (Biomarker, Beijing). Three SMAT cells (1–2 kb, 2–3 kb and 3–6 kb) were run on the PacBio RSII platform (NCBI accession number: SRP131708). The resulting library was sequenced using the Iso-Seq function of PacBio RS II systems (Pacific Biosciences, Menlo Park, CA, USA) [48].

For Illumina RNA-seq, equal RNA samples at each stage of each line, including three replicates, were collected. The purity and quality of the libraries was assessed using an Agilent 2100 Bioanalyzer (Agilent, Palo Alto, California, USA) and Qubit 2.0 (Invitrogen, Carlsbad, CA, USA). Then, the 18 libraries were sequenced using the Illumina HiSeqTM 2500 sequencing platform (Illumina Inc., San Diego, CA, USA) at Biomarker Technologies Corporation in Beijing. Subsequently, the iterative isoform-clustering (ICE) algorithm was used to cluster the full-length sequences from the same isoform. Similar full-length sequences were clustered together, and each cluster had a consistent sequence.

### Bioinformatics and differential expression analysis

For SMRT-seq, ROI sequences were extracted from raw reads and filtered for the cDNA primers and poly A tail. Then, the sequences were divided into full-length, non-full-length, chimeric and non-chimeric categories according to the presence of 3′-primer, 5′-primer and poly-A (optional). Using the Quiver algorithm to cluster non-full-length sequences, the consistent sequences were polished, and ones with high quality and low quality were obtained, respectively. The Illumina RNA-seq data were used to correct low-quality conformance sequences.

For Illumina RNA-seq, the raw RNA-seq reads were quality-checked using the FastQC (v0.11.8) to remove the adaptor sequences and low-quality reads [49]. The resulting high-quality clean reads were mapped independently to the reference genome of *R. communis* [1]. Bowtie (v2.2.3) was applied to modify the reference genome [50]. TopHat (v2.0.12) was used to align the paired-end clean reads to the reference genome [51].

Cufflinks (v2.1.1) was used to detect all transcripts using Quantitative Real-time Reverse Transcription PCR; [52]). Astalavista (v1.0) was applied to obtain the AS events [53]. LncTar is a tool for predicting the RNA targets of long noncoding RNAs [54]. The analysis of fusion genes and lncRNAs was performed with CPC, CNCI, CPAT and Pfam software, according to the respective instructions. TAPIS pipeline software [55] was used to identify APA.

### Differential expression analysis

The programs TopHat and Cufflinks were used to blast the sequencing reads against the reference genome of *R. communis* for the analyses of differential genes and transcript expression. It can evaluate the abundance of gene expression and also reveal new genes that have not been previously annotated using reference genomes [4]. The Fragments Per Kilobase of exon per Million fragments mapped (FPKM) method was used to calculate the abundance of gene expression. DESeq was used for analyzing biological duplicate samples obtained from DEG screening, and EBSeq [56] was used for non-biological duplicate samples. During the DEG screening, a false discovery rate (FDR) < 0.001 and fold change ≥8 were considered standard values. If the DEG fold change was ≥8, then a FDR < 0.001 was taken to indicate that the DEG was significantly different between the control and test group.

### Quantitative real-time reverse transcription PCR analysis

The transcript levels of ten candidate DEGs regulating seed weight were also verified by qRT-PCR. Total RNA (1 μg) was reverse transcribed using a reverse transcriptase (Takara, Japan). A 5 μl aliquot of 1:20 diluted cDNA was used as the template in a 20 μl PCR system. QRT-PCR was performed using SYBR Green qRT-PCR Master Mix (Kapa, china), encompassing an initial denaturation step at 95 °C for 5 min, followed by 40 cycles of denaturation at 95 °C for 15 s, annealing at 60 °C for 15 s, and extension at 72 °C for 32 s in an ABI PRISM7500 Sequence Detection System (Applied Biosystems, USA). The actin gene was used as the internal standard because it is uniformly expressed in *R. communis* tissues [57]. All reactions were performed using one biological sample with three technical replicates. The comparative Ct method [58] was used for the data analysis. Primers were designed using Primer 5.0 software (Premier, Canada), and the sequences are listed in Additional file 1: Table S11.

### Functional annotation

GO database was used to assign genes to broad functional categories. COG was used to classify gene products in an orthologous relationship. KOG (ftp://ftp.ncbi.nih.gov/pub/COG/KOG/kyva) database was used to classify the homologous into different orthology clusters by combining evolutionary relationships. KEGG database was used to assign enzymes to known pathways. Pfam database was used for protein domain annotation. Overall, the functional annotation analysis of novel and differentially expressed genes was conducted by performing a blast search against the GO, COG, KEGG pathway, SwissProt and NR databases using BLAST software.

## Additional files

Additional file 1: **Table S1.** Statistics of sequencing data for three SMRT cells (1–2 k, 2-3 k and 3-6 k) by PacBio SMRT sequencing. **Table S2.** Statistics of reads of insert (ROI) for three SMRT cells (1–2 k, 2-3 k and 3-6 k) by PacBio SMRT sequencing. **Table S3.** Statistics of full-length reads for three SMRT cells (1–2 k, 2-3 k and 3-6 k) by PacBio SMRT sequencing. **Table S4.** Statistics of iterative clustering for error correction (ICE) for all isoforms by PacBio SMRT analysis. **Table S5.** The gene structure optimization information by PacBio SMRT sequencing. **Table S6.** The annotated information of 21,122 novel genes. **Table S7.** Detailed information on 250 fusion genes. **Table S8.** The list of 1356 TF genes. **Table S9.** Statistics of sequencing data for strains 349 and 1115 at three time points (7 DAF, 14 DAF and 21 DAF) by Illumina RNA sequencing. **Table S10.** Comparison of transcriptome sequencing reads with genome references by Illumina RNA sequencing. **Table S11.** The primers for quantitative real-time PCR in this study. (ZIP 1535 kb)

Additional file 2: **Figure S1.** Correlation thermograms between 18 samples from Illumina RNA sequencing. **Figure S2.** ROI read length distribution of each size (1–2 k, 2-3 k and 3-6 k) of bins in the cDNA database. **Figure S3.** GO classification according to cellular component, molecular function and biological process of differentially expressed genes. Strain 349 vs. strain 1115 at 7 DAF (**A**), 14 DAF (**B**), and 21 DAF (**C**). **Figure S4.** KEGG classification and Pathway enrichment statistics of differentially expressed genes. (**A**) KEGG classification between strains 349 and 1115 at 7 DAF. (**B**) KEGG classification between 349 and 1115 at 14 DAF. (**C**) KEGG classification between 349 and 1115 at 21 DAF. (**D**) Pathway enrichment statistics between 349 and 1115 at 7 DAF. (**E**) Pathway enrichment statistics between 349 and 1115 at 14 DAF. (**F**) Pathway enrichment statistics between 349 and 1115 at 14 DAF. **Figure S5.** COG function classification of differentially expressed genes. (**A**) 349 vs. 1115 at 7 DAF. (**B**) 349 vs. 1115 at 14 DAF. (**C**) 349 vs. 1115 at 21 DAF. (ZIP 29130 kb)

## Data Availability

Data for this study will be available upon publication of the manuscript; until then, the raw data can be made available to researchers upon reasonable request to the corresponding author.

## Abbreviations

AAT: Oleoyl-ACP thioesterase
ACS: Acyl-CoA synthase
APA: Alternative polyadenylation
AS: Alternative splicing
BLAST: Basic local alignment search tool
CCS: Circular-consensus
CNCI: Coding-Non-Coding Index
COG: Cluster of orthologous groups of proteins
CPAT: Coding Potential Assessment Tool
CPC: Coding potential calculator
DAF: Days after flowering
DAG: Diacylglycerol
DAGT: Diacylglycerol acyltransferase
FA: Hydroxy fatty acid
FAH12: Oleoyl-12-hydroxylase
FDR: False discovery rate
FL: Full-length
FPKM: Fragments Per Kilobase of exon per Million fragments mapped
GO: Gene ontology
GPAT: Glycerol-3-phosphate acyltransferase
ICE: Iterative isoform-clustering
KEGG: Kyoto Encyclopedia of Genes and Genomes
KOG: Eukaryotic ortholog groups
LACS: Long-chain lipoyl-CoA synthetase
LncRNAs: Long non-coding RNAs
LPAAT: Lysophosphatidic acid acyltransferase
LPC: Lysophosphatidylcholine
LPCAT: Lysophosphatidylcholine acyltransferase
NGS: Next-generation sequencing
NR: Non-redundant protein
PA: Phosphatidic acid
PAP: Phosphatidic acid phosphatase
PC: Phosphatidylcholine
Pfam: Protein family
PLA2: Phospholipase A2
PLC2: Phospholipase C2
QRT-PCR: Quantitative real-time reverse transcription PCR
R: Ricinoleate
ROI: Reads of insert
RRR: Triricinolein
RRRR: Diricinoleoylglycerol
SMRT: Single-molecule real-time
Swiss-Prot: The manually annotated and reviewed protein sequence database
TFs: Transcription factors
UTR: Untranslated region
