# Supplementary figures and images for: A survey of transcriptome complexity using PacBio single-molecule real-time analysis combined with Illumina RNA sequencing for a better understanding of ricinoleic acid biosynthesis in Ricinus communis
Source: BMC Genomics. 2019 Jun 6;20:456. doi: 10.1186/s12864-019-5832-9 (PMC6555039; doi:10.1186/s12864-019-5832-9)

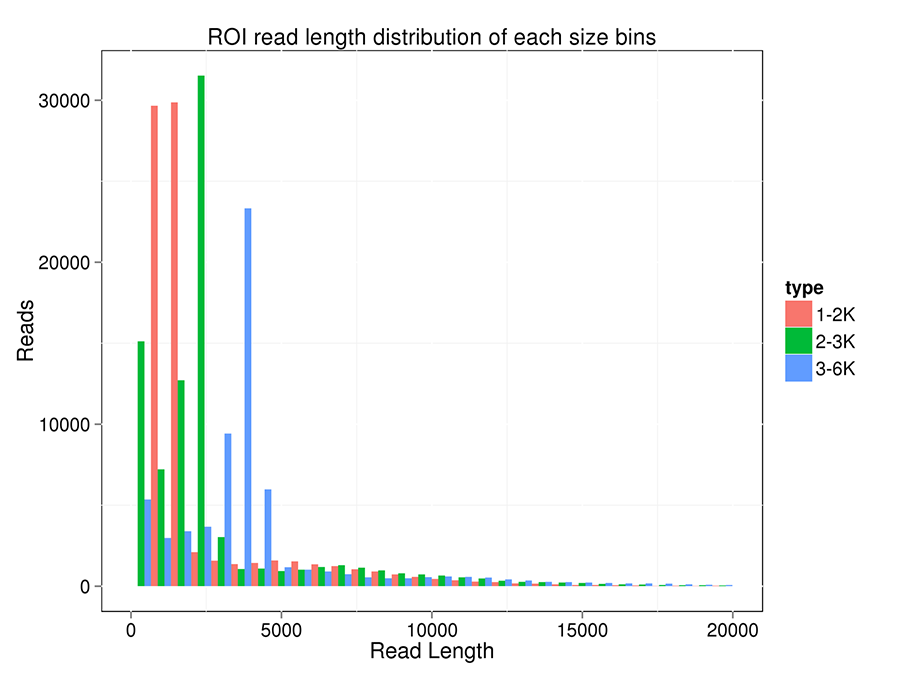

Supplement: Supplementary file 2 — Figure S1. Correlation thermograms between 18 samples from Illumina RNA sequencing. Figure S2. ROI read length distribution of each size (1–2 k, 2-3 k and 3-6 k) of bins in the cDNA database. Figure S3. GO classification according to cellular component, molecular function and biological process of differentially expressed genes. Strain 349 vs. strain 1115 at 7 DAF (A), 14 DAF (B), and 21 DAF (C). Figure S4. KEGG classification and Pathway enrichment statistics of differentially expressed genes. (A) KEGG classification between strains 349 and 1115 at 7 DAF. (B) KEGG classification between 349 and 1115 at 14 DAF. (C) KEGG classification between 349 and 1115 at 21 DAF. (D) Pathway enrichment statistics between 349 and 1115 at 7 DAF. (E) Pathway enrichment statistics between 349 and 1115 at 14 DAF. (F) Pathway enrichment statistics between 349 and 1115 at 14 DAF. Figure S5. COG function classification of differentially expressed genes. (A) 349 vs. 1115 at 7 DAF. (B) 349 vs. 1115 at 14 DAF. (C) 349 vs. 1115 at 21 DAF. (ZIP 29130 kb) [file 12864_2019_5832_MOESM2_ESM.zip › Figure S1.tif]

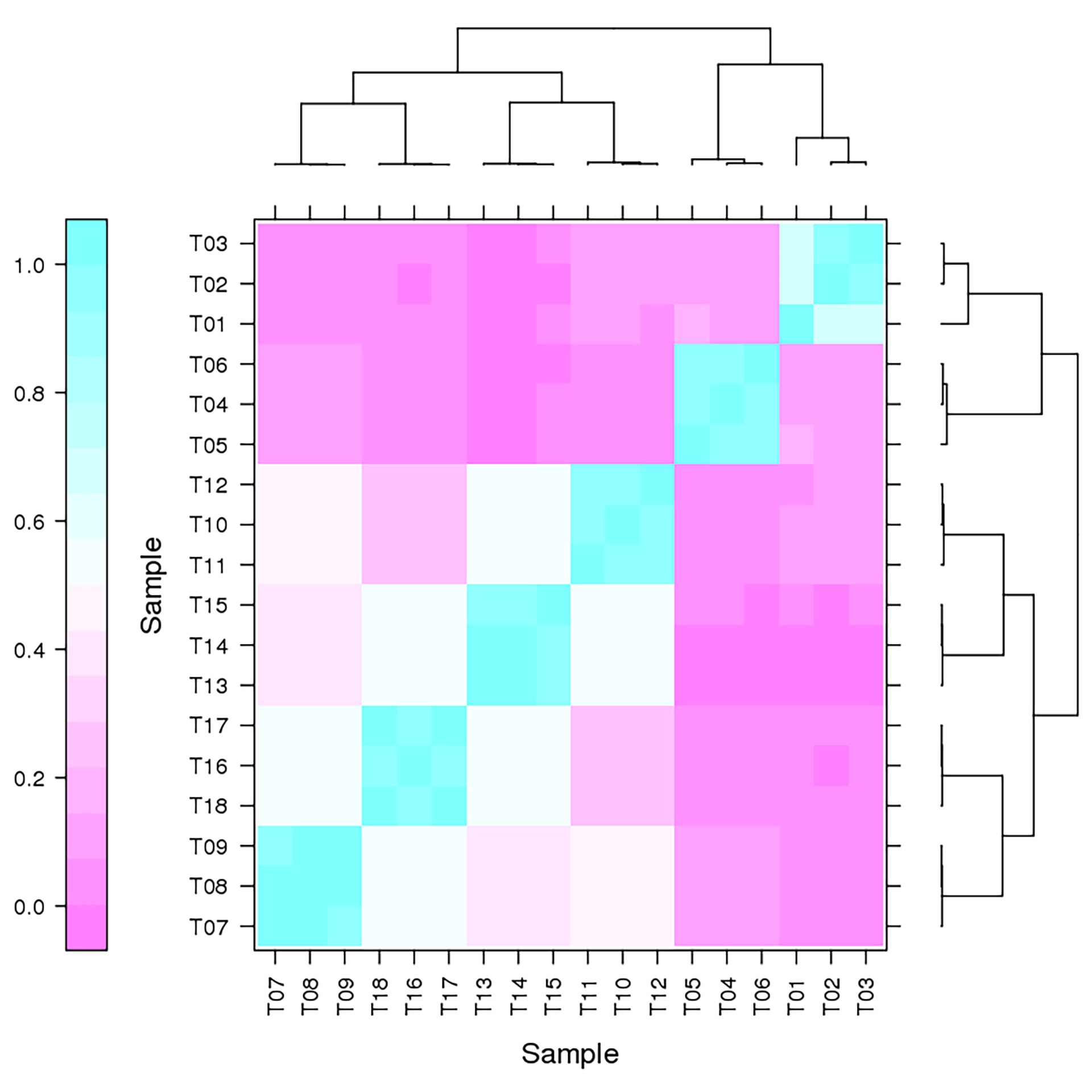

Supplement: Supplementary file 2 — Figure S1. Correlation thermograms between 18 samples from Illumina RNA sequencing. Figure S2. ROI read length distribution of each size (1–2 k, 2-3 k and 3-6 k) of bins in the cDNA database. Figure S3. GO classification according to cellular component, molecular function and biological process of differentially expressed genes. Strain 349 vs. strain 1115 at 7 DAF (A), 14 DAF (B), and 21 DAF (C). Figure S4. KEGG classification and Pathway enrichment statistics of differentially expressed genes. (A) KEGG classification between strains 349 and 1115 at 7 DAF. (B) KEGG classification between 349 and 1115 at 14 DAF. (C) KEGG classification between 349 and 1115 at 21 DAF. (D) Pathway enrichment statistics between 349 and 1115 at 7 DAF. (E) Pathway enrichment statistics between 349 and 1115 at 14 DAF. (F) Pathway enrichment statistics between 349 and 1115 at 14 DAF. Figure S5. COG function classification of differentially expressed genes. (A) 349 vs. 1115 at 7 DAF. (B) 349 vs. 1115 at 14 DAF. (C) 349 vs. 1115 at 21 DAF. (ZIP 29130 kb) [file 12864_2019_5832_MOESM2_ESM.zip › Figure S2.tif]

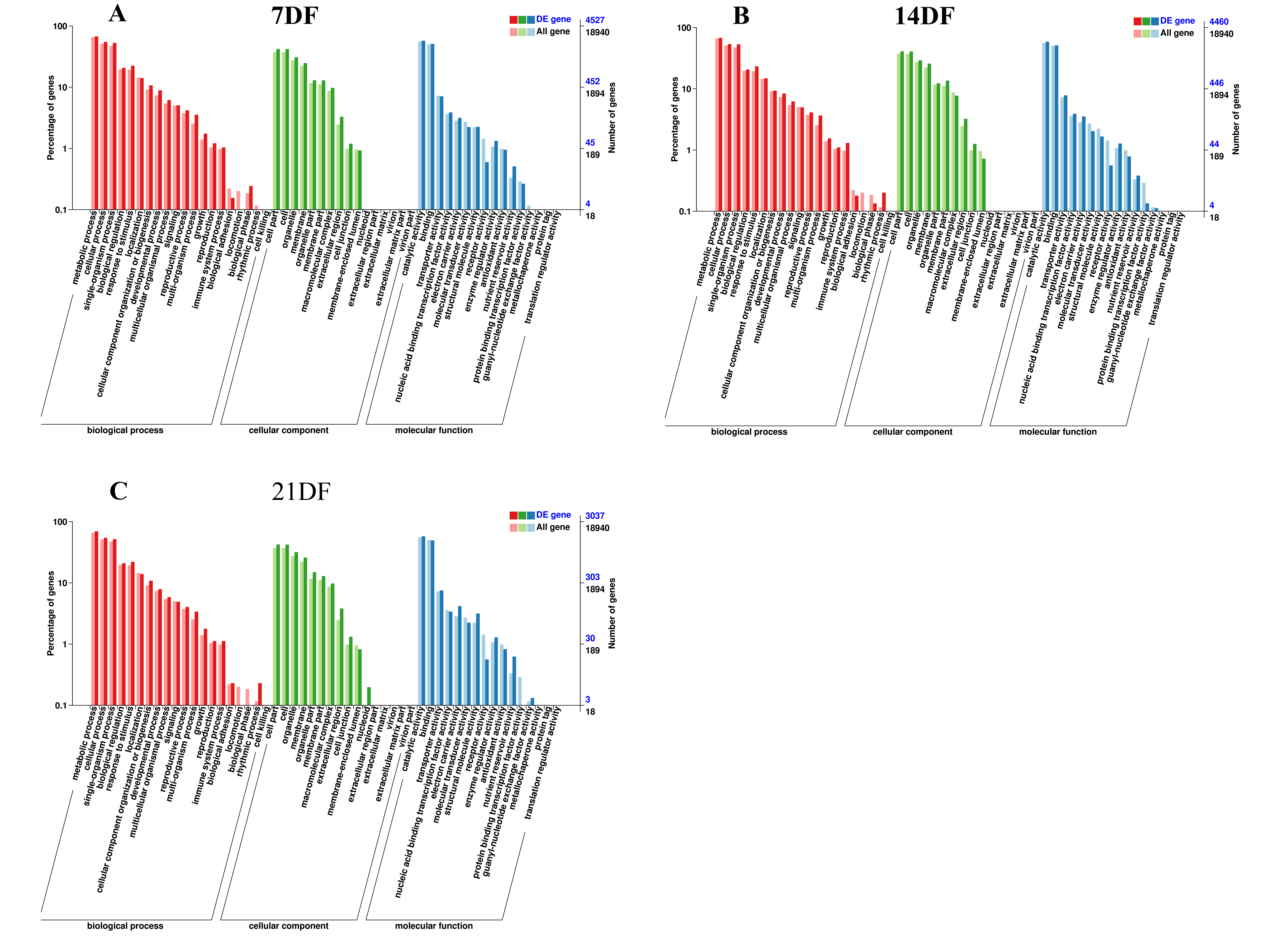

Supplement: Supplementary file 2 — Figure S1. Correlation thermograms between 18 samples from Illumina RNA sequencing. Figure S2. ROI read length distribution of each size (1–2 k, 2-3 k and 3-6 k) of bins in the cDNA database. Figure S3. GO classification according to cellular component, molecular function and biological process of differentially expressed genes. Strain 349 vs. strain 1115 at 7 DAF (A), 14 DAF (B), and 21 DAF (C). Figure S4. KEGG classification and Pathway enrichment statistics of differentially expressed genes. (A) KEGG classification between strains 349 and 1115 at 7 DAF. (B) KEGG classification between 349 and 1115 at 14 DAF. (C) KEGG classification between 349 and 1115 at 21 DAF. (D) Pathway enrichment statistics between 349 and 1115 at 7 DAF. (E) Pathway enrichment statistics between 349 and 1115 at 14 DAF. (F) Pathway enrichment statistics between 349 and 1115 at 14 DAF. Figure S5. COG function classification of differentially expressed genes. (A) 349 vs. 1115 at 7 DAF. (B) 349 vs. 1115 at 14 DAF. (C) 349 vs. 1115 at 21 DAF. (ZIP 29130 kb) [file 12864_2019_5832_MOESM2_ESM.zip › Figure S3.tif]

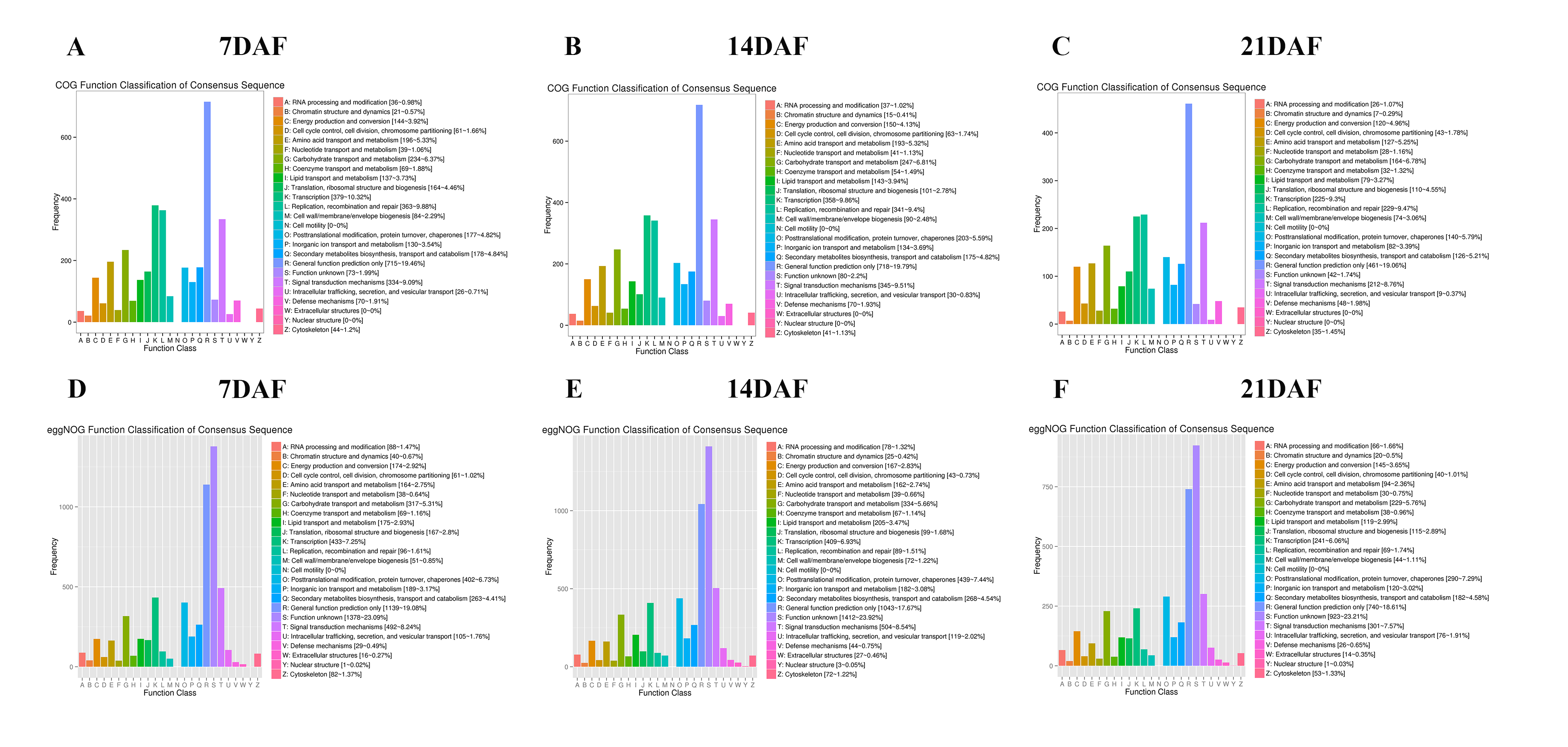

Supplement: Supplementary file 2 — Figure S1. Correlation thermograms between 18 samples from Illumina RNA sequencing. Figure S2. ROI read length distribution of each size (1–2 k, 2-3 k and 3-6 k) of bins in the cDNA database. Figure S3. GO classification according to cellular component, molecular function and biological process of differentially expressed genes. Strain 349 vs. strain 1115 at 7 DAF (A), 14 DAF (B), and 21 DAF (C). Figure S4. KEGG classification and Pathway enrichment statistics of differentially expressed genes. (A) KEGG classification between strains 349 and 1115 at 7 DAF. (B) KEGG classification between 349 and 1115 at 14 DAF. (C) KEGG classification between 349 and 1115 at 21 DAF. (D) Pathway enrichment statistics between 349 and 1115 at 7 DAF. (E) Pathway enrichment statistics between 349 and 1115 at 14 DAF. (F) Pathway enrichment statistics between 349 and 1115 at 14 DAF. Figure S5. COG function classification of differentially expressed genes. (A) 349 vs. 1115 at 7 DAF. (B) 349 vs. 1115 at 14 DAF. (C) 349 vs. 1115 at 21 DAF. (ZIP 29130 kb) [file 12864_2019_5832_MOESM2_ESM.zip › Figure S5.tif]
